# Microstructure of a Model Fresh Cheese and Bioaccessibility of Vitamin D_3_ Using In Vitro Digestion

**DOI:** 10.3390/gels5010016

**Published:** 2019-03-10

**Authors:** Nuria Castaneda, Youngsoo Lee

**Affiliations:** Department of Food Science and Human Nutrition, University of Illinois, Urbana-Champaign, Champaign, IL 61801, USA; nuria.castaneda@hotmail.com

**Keywords:** microstructure, soft solids, model fresh cheese, bioaccessibility, vitamin D_3_

## Abstract

In this study, the effect of a composition (protein to fat (P/F) ratio) and a processing condition (homogenization pressure for emulsification of cheese milk) on the texture, microstructure, and bioaccessibility of vitamin D_3_ of a model acid coagulated fresh cheese was evaluated. It was hypothesized that increasing P/F ratios (0.9, 1.3, 1.7, and 2) and homogenization pressures (17, 50, 75, and 150 MPa) will decrease the particle size of the cheese milk emulsion. The decreased emulsion particle size will result in a more rigid and elastic cheese matrix with smaller pore sizes, with an increased interfacial surface area of fat particles, which will then improve the bioaccessibility of vitamin D_3_. The P/F ratio exhibited a positive impact on the texture in a large deformation analysis. On the other hand, the effect of the P/F ratio and homogenization pressure was not significant on rheological properties of the cheese using a small deformation by means of a frequency sweep test, nor the porosity determined by environmental scanning electron microscopy (ESEM). These results suggested that the modification of the microstructure of acid coagulated fresh cheeses required other variables than P/F ratio and homogenization pressure probably due to a compression step after curd formation. Interestingly, the bioaccessibility of vitamin D_3_ measured by in vitro digestion was reduced as P/F ratio and homogenization pressure increased, which may indicate a reinforced protein–protein interaction that affected protein hydrolysis.

## 1. Introduction

The interactions between food components at micro- and nano-scales dictate the physical, chemical, and nutritional characteristics of a processed food product. The need to manufacture food products with health benefits by using separated materials to form constituted matrices after the application of processing [1] is becoming more critical for the food industry and consumers than ever.

Several studies have revealed the effects of processing on food microstructure, nutrient digestion, and absorption, and many of these studies have focused on minimally processed food matrices [2,3,4]. It is important to evaluate complex food systems to understand the links between food composition, processing, and nutrient disposition to design food products that will deliver specific functionalities.

To recreate complex food systems, the use of proteinaceous matrices and emulsions are considered appropriate to assess structure at the micro- and nano-scales due to their versatility on structure formation [1,5,6,7]. Milk proteins and milk protein gelation have been intensively investigated due to their ability for imparting structure, texture, flavor, and functionality [8,9]. In cheese matrices, aggregated casein micelles create a microstructural network with entrapped solid fat globules and serum [10], and their digestion is directly related to their physical characteristics, specifically textural and structural properties [11]. Also, it has been demonstrated that milk proteins are good vehicles to deliver liposoluble micronutrients, since they use several mechanisms to bind hydrophobic molecules [12].

In this study, we have considered the use of an acid coagulated model fresh cheese to evaluate the effects of composition (protein to fat (P/F) ratio) and a processing condition (homogenization pressure for emulsification of cheese milk) as two variables that can modify microstructure [13,14,15,16,17,18]. The structure and microstructure of the cheese was analyzed using large and small deformation techniques as well as imaging analysis. Subsequently, we evaluated the bioaccessibility of vitamin D_3_ due to the two variables using two-stage in vitro digestion.

## 2. Results and Discussion

### 2.1. Particle Size

**Figure 1:**
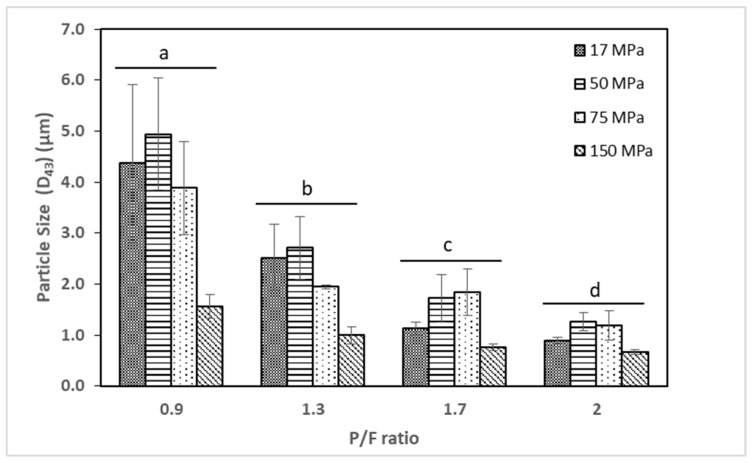
Particle size $\left(d_{43}\right)$ of cheese milk emulsion in µm. As protein to fat (P/F) ratio and homogenization pressure increased, particle size of the cheese milk emulsion tended to decrease. Results are expressed as mean ± standard deviation (*n* = 3). Statistical differences are denoted by different letters (**a**–**d**) for each level of protein to fat ratio (*p* < 0.05).

Particle size analysis of the cheese milk emulsion showed that the particle size $\left(d_{43}\right)$ decreased as the P/F ratio increased (p<0.05) and as the homogenization pressure increased (p<0.05), suggesting the overall trend of the formation of smaller fat droplets due to high levels of pressure and a higher amount of milk proteins avoiding fat droplets to coalescence, as shown in Figure 1 [19,20]. In relation to homogenization pressure, despite the fact that samples processed at 17 and 50 MPa are statistically not different (p>0.05), the samples processed at 17 MPa resulted in a smaller particle size compared to those at 50 MPa, especially at P/F ratios above 1.7. It may be because the excess protein formed an additional layer on the emulsion at 50 MPa.

### 2.2. Texture Profile Analysis

**Figure 2:**
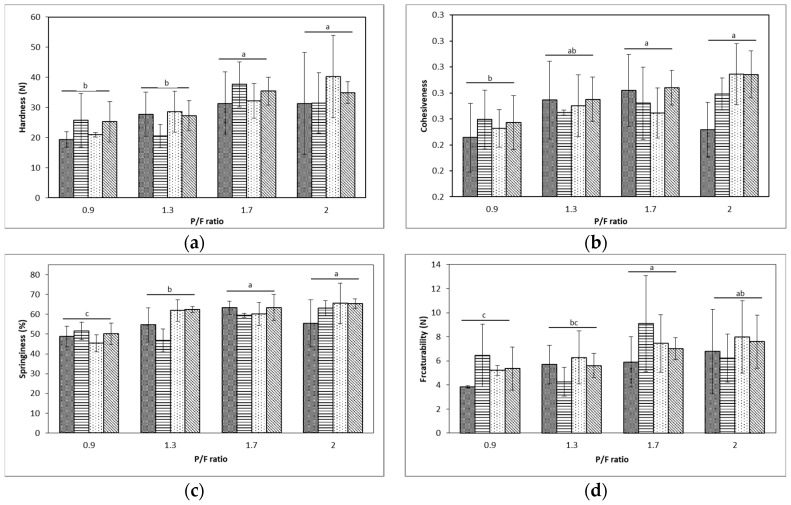
Texture properties of model fresh cheese samples. Statistical differences are denoted by different letters for each level of protein to fat ratio (*p* < 0.05). Bars within groups (17, 50, 75, and 159 MPa, respectively) did not differ significantly. Formulation affected texture properties of hardness (**a**), cohesiveness (**b**), springiness (**c**), and fracturability (**d**).

P/F ratio in the model fresh cheese demonstrated having a major impact on textural parameters obtained using a large deformation test. The samples processed at a higher P/F ratio obtained higher hardness, cohesiveness, springiness, and fracturability (p<0.05), as shown in Figure 2, indicating that the increased volume fraction of protein [21] created a dense and strong network between caseins [22,23] and thus a firmer texture. At a 0.9 P/F ratio, the presence of greater amounts of fat resulted in the significantly (p<0.05) lower hardness, cohesiveness, and springiness. The low textural parameters indicated that the weak structure of the sample was attributed to higher fat retention, allowing for fat globules to occupy free spaces within the porous matrix. Some of the fat globules might act as fillers and become incompatible with the protein matrix [24,25,26].

Different levels of homogenization pressure did not show a significant effect on the textural parameters (p>0.05) for the model fresh cheese. Although no statistical significance was found, there were visible differences between samples’ curd formation at lowest and highest homogenization pressures. When processed at higher pressures (150 MPa), a shattered curd was observed as described in other studies where weak milk gels have been formed when using microfluidization at 170 MPa [27], however further processing factors such as pressing might have minimized the effect of homogenization pressure on textural properties.

### 2.3. Small Amplitude Oscillatory Shear

**Figure 3:**
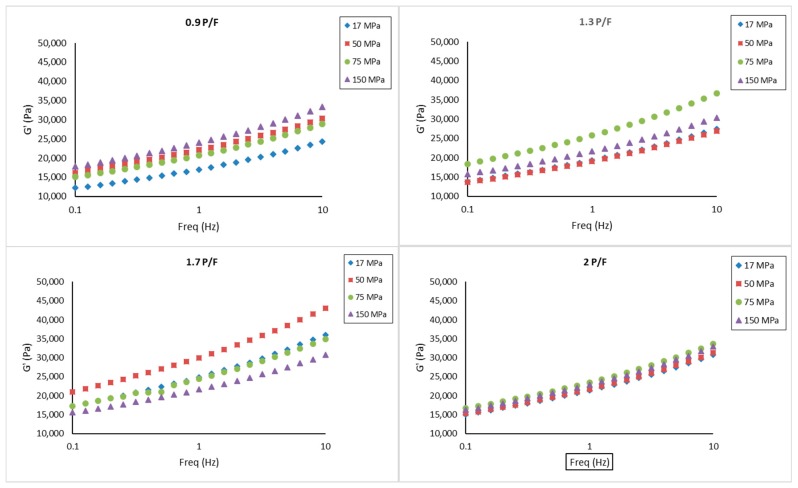
Storage modulus (G′) spectra for model fresh cheese samples processed at different protein to fat ratios (0.9, 1.3. 1.7, and 2) and homogenization pressures (17, 50, 75, and 150 MPa). Results expressed as mean (*n* = 3).

**Figure 4:**
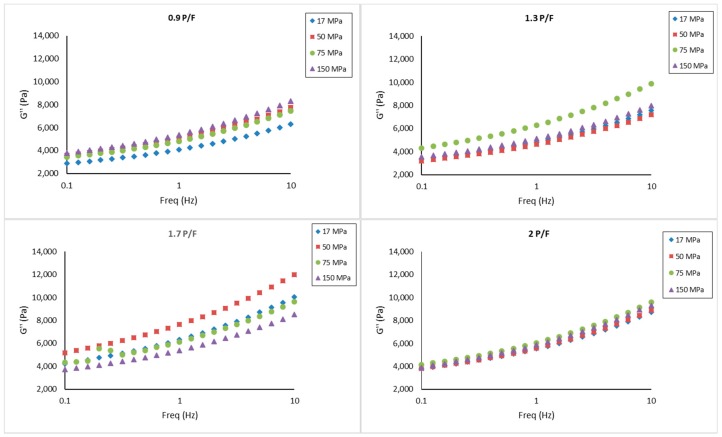
Loss modulus (G″) spectra for model fresh cheese samples processed at different protein to fat ratios (0.9, 1.3. 1.7, and 2) and homogenization pressures (17, 50, 75, and 150 MPa). Results expressed as mean (*n* = 3).

**Figure 5:**
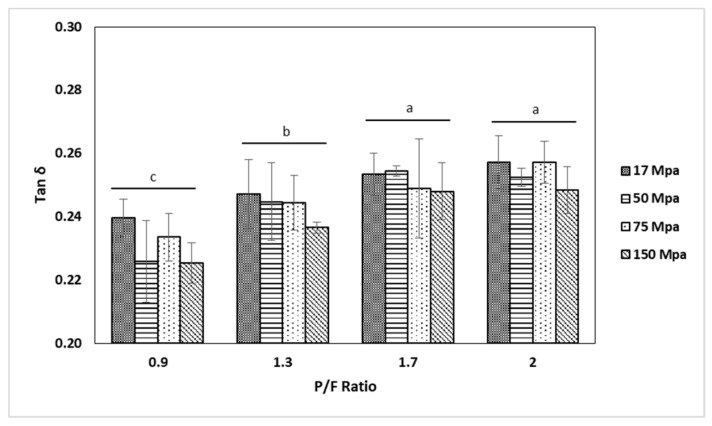
Tan δ as a function of protein to fat ratio (P/F). Results expressed as mean ± standard deviation (*n* = 3). Statistical differences are denoted by different letters for each level of P/F ratio (*p* < 0.05). Bars within groups did not differ significantly.

The results from a frequency sweep test on the cheeses showed for all samples that storage modulus (G′) was higher compared to loss modulus (G′′), indicating dominant elastic characteristics, as shown in Figure 3 and Figure 4. However, no clear trend was observed due to the P/F ratio and the homogenization pressure. Sanchez et al. [28] reported that smaller sizes of milk fat globules formed by higher homogenization pressures in cheese milk, resulted in strong structures in a model acid fresh cheese, and high levels of fat also created a firmer and elastic cheese due to recombined fat globules assimilated as pseudo-proteins. The difference could be due to the variation in processing temperature as well as acidification and pressing method used. On the other hand, Cobos et al. [29] concluded that the rheological properties of recombined acid milk gels were not significantly affected by fat content nor homogenization pressures using a microfluidization process. Cobos et al. [30] pointed out that the effect of heat treatment and solid levels were more prominent than homogenization pressure in rheological properties of acidified milk gels. All tan δ values were less than 1 (between 0.21 and 0.28), indicating a viscoelastic behavior of gels. Interestingly, by analyzing samples at 1 Hz, tan δ was significantly smaller (p<0.05) at P/F of 0.9 and 1.3 than 1.7 and 2, as shown in Figure 5. The assumption of a higher amount of fat globules acting as pseudo-proteins and improving the protein interactions amongst the aggregates [28] might apply here, however this explanation needs further study. The complex viscosity (η*) showed no significant differences based on formulation or homogenization pressure.

When analyzing these results with texture profile analysis (TPA) data, where hardness, cohesiveness, springiness, and fracturability significantly increased at higher levels of P/F ratios, suggest that fat and protein had a major effect at large deformations compared to its effect at small deformations, and the homogenization pressure effect might be hindered by the solid content and the pressing step in cheese preparation.

### 2.4. Microstructure: Porosity and Pore Size

**Figure 6:**
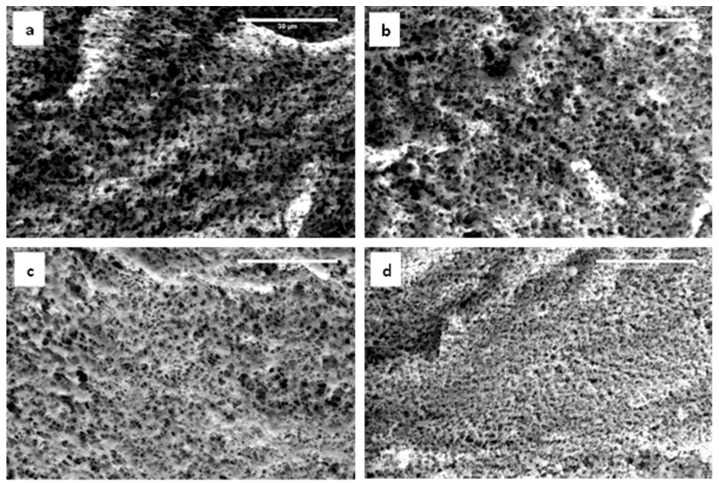
Environmental scanning electron microscopy (ESEM) micrographs at 2000× processed at 150 MPa. (**a**) 0.9, (**b**) 1.3, (**c**) 1.7, and (**d**) 2 P/F ratios, respectively. Scale bar at 30 µm.

Pore size and porosity of the model fresh cheeses were analyzed using the images (*n* = 3) taken by environmental scanning electron microscopy (ESEM). The porosity of samples ranged from 49.8% and 67.9%. The porosity and pore size in volume did not show statistical differences between samples (p>0.05). It was inferred from this result that the pore formation between casein aggregates was affected by the drainage of whey during 15 h of curd pressing [31]. From ESEM images, the average size of pores varied around 0.57 µm in diameter. Although the effect of formulation and homogenization pressure on the pore size was not significant (p>0.05), when comparing images at lowest and highest homogenization pressures, as shown in Figure 6, the differences in structure were observed at the highest P/F ratio, showing a dense network of aggregated caseins with smaller overall pores, probably due to the higher protein fraction.

Given the results in pore size and porosity, the approach to modify the microstructure of acid coagulated fresh cheese may need to include other variables such as the type of acidulant, the temperature of coagulation, and the drainage of whey [31,32].

### 2.5. Fortification of Model Fresh Cheese with Vitamin D_3_

Three of the model fresh cheese samples were selected for vitamin D_3_ fortification. The samples P1R1 (17 MPa—0.9 P/F ratio), P1R4 (17 MPa—2 P/F ratio), and P4R4 (150 MPa—2 P/F ratio) were selected from the lowest to highest homogenization pressures and P/F ratios, respectively.

Vitamin D_3_ concentration was analyzed during all the processing steps of cheese preparation to determine its stability, as shown in Table 1. The amount of vitamin D_3_ added in the cheese milk samples did not change after microfluidization and heat treatment (p>0.05), indicating the vitamin was stable during processing. The amount of vitamin D_3_ in the cheese milk decreased as the P/F ratio and homogenization pressure increased, which might indicate a lower extraction efficiency for the samples containing higher amounts of protein and smaller sizes of emulsion particles. The same quantity of vitamin D_3_ was added to all cheese milk samples, but the state of the emulsified matrix containing smaller particle sizes at higher P/F ratios and homogenization pressures created a very stable interface of caseins and whey proteins [33,34] that might have hindered the extraction of vitamin D_3_ from the lipid phase. The amount of vitamin D_3_ in the model cheese was not significantly different between the samples. The soft gel formation during the curd process changed the protein network at the interface and the compression process resulted in the concentration of vitamin D_3_ in the model cheese.

### 2.6. Bioaccessibility by In Vitro Digestion

The bioaccessibility of vitamin D_3_ was analyzed as the percentage of vitamin D_3_ recovered in the micellar phase after in vitro digestion compared to the amount of vitamin D_3_ found in the model fresh cheese.

**Figure 7:**
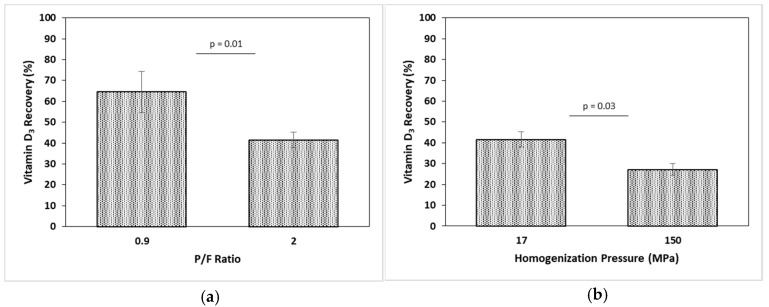
Effect of protein to fat (P/F) ratio (**a**) and homogenization pressure (**b**) on vitamin D_3_ bioaccessibility (vitamin D_3_ recovery after the in vitro digestion). Differences between samples analyzed at 0.9 and 2 P/F, and 17 and 150 MPa are shown by an independent t-test. Results expressed as mean ± standard deviation (*n* = 3).

Results showed a significant effect (p<0.05) of the P/F ratio and homogenization pressure in the vitamin D_3_ bioaccessibility, as shown in Figure 7; the samples processed at higher P/F (2 P/F) ratio and homogenization pressure (150 MPa) had lower vitamin D_3_ recovery in the micellar phase. Given that all samples contained a similar amount of vitamin D_3,_ this result might indicate lower protein hydrolysis during digestion, thus a partial protein degradation might prevent release of the liposoluble fraction [35,36]. On the other hand, the association and arrangement of acid coagulated gels from recombinant homogenized milk has been shown to affect microstructure; during acidification, fat globules are replaced by micellar caseins and denatured whey proteins, and a layer around the fat globules is adsorbed. Higher concentration of denatured whey proteins has been shown to provide more cross-links in the structure, making a stronger interaction in the protein network [25,37,38]. The formation of a strong protein network is enhanced by homogenization and also by the protein load (mg protein/m^2^ fat surface) [37,39]. The higher homogenization pressures and P/F ratio reinforced the structure of the model fresh cheese and lowered the protein hydrolysis during the in vitro digestion. However, this hypothesis needs a further investigation of the microstructure the cheese since rheology and porosity data for the model fresh cheese was not conclusive.

## 3. Conclusions

The model fresh cheese elaborated with various P/F ratios and homogenization pressures was studied in order to describe structural changes in the cheese matrix and its impact on vitamin D_3_ bioaccessibility. The effect of the P/F ratio in the formulation was evident in a large deformation test using TPA when structural bonds were broken. The effect of the P/F ratio was not observed by a small deformation test using an oscillatory frequency sweep, indicating additional tests may be needed to assess the structural properties of an acid coagulated fresh cheese. Similarly, the P/F ratio did not show a significant trend in porosity and pore size, probably due to 15 h of curd pressing and a similar moisture content that was retained in all samples. Homogenization pressure did not affect the structural parameters in the model fresh cheese, although the changes in casein micelles have been shown with microfluidization in the past [39], when gels were treated with rennet and not acid. The conformation of aggregated proteins is more complex in acid induced gels [40,41] and this might minimize the effect of homogenization pressure in textural and rheological properties. Interestingly, both the P/F ratio and homogenization pressure did affect the bioaccessibility of vitamin D_3_. As P/F ratio and homogenization pressure increased, the bioaccessibility of vitamin D_3_ decreased, suggesting higher interconnectivity of the protein matrix and the smaller fat particle size within the casein aggregates leading to lower hydrolysis during digestion, impeding the release of fat soluble components. A further study is required to better understand the relationship between the microstructure of cheese matrix and the bioaccessibility of vitamin D_3_.

Microstructure of complex soft solids needs additional investigation since many factors regarding constituents, as well as processing parameters, interact together to influence the micro- and macro-structural characteristics. Understanding the critical factors will help to deliver complex food systems with enhanced nutritional properties and provide new and functional food products to consumers.

## 4. Materials and Methods

### 4.1. Preparation of Model Fresh Cheese

Model fresh cheese was prepared in a similar means to “Queso Blanco” [24,42] with variations in formulation and microfluidization pressures. Four levels of protein to fat (P/F) ratio and four levels of microfluidization pressure, as shown in Table 2, were selected from preliminary studies using a range of P/F ratios that could be processed using the microfluidization technology. All formulations were prepared with the same number of solids. The experiments were replicated three times for textural, rheological, and image analysis.

Low-heat skim milk powder (34% protein, SMP, MP Biomedicals, LLC, Santa Ana, CA, USA) and anhydrous milk fat (99.9% fat, AMF, Danish Maid Butter Co., Chicago, IL, USA) were used to adjust the P/F ratio of the samples. The designated amount of SMP was reconstituted in deionized water at 60 °C [43] and mixed in a professional mixer (KitchenAid^®^ Professional 5Q Mixer, KitchenAid, Benton Harbor, MI, USA) at medium shear until completely dispersed. Melted AMF at 40 °C was added in a pre-homogenization step using an IKA T-25 Digital High-Speed Homogenizer (IKA Works Inc., Wilmington, NC, USA) at 8000 rpm. Rotational speed was brought up to 11,000 rpm for additional mixing for 5 min. Samples were microfluidized using an M-110P microfluidizer (Microfluidics^TM^, Westwood, MA, USA) equipped with a Y-chamber. Cheese milk emulsion was kept under refrigeration at 4 °C for approximately 6 h until cheese preparation. Cheese curd was formed using heat-induced coagulation [42,44,45] by heating up the emulsion at 82 °C and 0.02 *w*/*w* % of calcium chloride was added [46]. Emulsion was acidified with 1:10 glacial acetic acid (AFCO, Chambersburg, PA, USA) reaching a pH between 5.0 and 5.2 [47,48]. After the curd formation, 2.5 *w*/*w* % of sodium chloride (crystalline/certified ACS, Fisher Scientific, Fair Lawn, NJ, USA) was added [42,49,50]. Cheese curd was drained and pressed overnight (15 h) with a Deluxe Dutch Cheese Press equipped with a 4.5 kg weight (~3.5 kPa, The Sausage Maker Inc., Buffalo, NY, USA).

After pressing, moisture content of each model fresh cheese was measured using a Mettler Toledo Moisture Analyzer HB43-S (Mettler Toledo International Inc., Columbus, OH, USA). For all samples, the moisture content did not show significant differences (p>0.05) due to the standardized solids content.

### 4.2. Particle Size Analysis of Cheese Milk Emulsion

After microfluidization, duplicates of 20 mL were obtained from each sample for particle size analysis ($d_{43}$). Average particle size was measured by a Shimadzu SALD-2300 Laser Diffraction Particle Size Analyzer equipped with a SALD-MS23 sampler (Shimadzu Scientific Instruments, Inc., Columbia, MD, USA) using a refractive index of 1.45 ± 0.02 [51].

### 4.3. Textural and Rheological Characteristics of Model Fresh Cheese

#### 4.3.1. Texture Profile Analysis (TPA) of Model Fresh Cheese

Textural properties of the model fresh cheese were analyzed using a texture profile analysis with a double compression test. Four samples were taken from each replicate and cut into 2 × 2 × 2 cm cubes with a carbon sharp blade [52]. The test was performed at 25 °C using TA.XT. Plus Texture Analyzer (Stable Micro Systems LTD., Vienna Court, UK) with a 5 kg loading cell attached to a 50 mm compression plate; speed was set up to 1 mm/s and 75% strain [18,22,53,54]. Hardness, cohesiveness, springiness, and fracturability were recorded.

#### 4.3.2. Small Amplitude Oscillatory Shear

Rheological moduli were analyzed using an ARES-G2 oscillatory rheometer equipped with an Advanced Peltier System, and TRIOS^®^ software (V3.0, TA Instruments, New Castle, DE, USA). A 25 mm stainless steel serrated parallel top plate was used at a constant temperature of 25 °C. Samples were cut into a round shape with a 30 mm diameter plain edge round cutter and equilibrated at room temperature (25 °C) for one hour prior to the analysis. The linear viscoelastic region (LVR) was determined by dynamic strain sweep at a constant frequency of 10 Hz with a strain range between 0.1 and 1%. The frequency sweep test was conducted from 0.1 to 10 Hz [55] at a 0.5% constant strain. Elastic and viscous moduli (G′ and G″), complex viscosity (η*), and tan δ were obtained.

### 4.4. Microstructure: Porosity and Pore Size

Environmental scanning electron microscope model Phillips XL30 ESEM-FEG (FEI Company, Waltham, MA, USA) was used to evaluate porosity and pore size of model fresh cheeses. Sample pieces of 3 × 2 × 10 mm were cut with a razor blade and kept at 4 °C in sealed containers for one hour before analysis [48]. Samples were frozen in liquid nitrogen for about 3–4 min fractured and mounted on the stage. Images were taken at 1 Torr wet mode with a Peltier stage, observed at 20 kV and 2000× magnification. Porosity and pore size were analyzed using MATLAB (version 7.0.4.356 R14, The MathWorks Inc., Natick, MA, USA) and the MATLAB Processing Toolbox (MathWorks 2014) using the grey scale thresholding method described by Kuo and Lee [56].

### 4.5. Vitamin D_3_ Fortification of Model Fresh Cheese

#### 4.5.1. Defining Standard Concentration and Standard Curve

A standard concentration of vitamin D_3_ was used to generate the standard curve for HPLC. To determine the standard concentration, a primary stock solution of vitamin D_3_ was prepared to a concentration of 20 µg/mL of ethanol. The exact concentration of the stock solution was determined by its extinction coefficient at 260 nm for cholecalciferol in ethanol ($E^{1\%}$ = 475) [57]. Five replicates of 1 mL were filtered in 0.45 µm PTFE (Macherey-Nagel Inc., Bethlehem, PA, USA) and analyzed in a GENESYS 10S UV-Vis Spectrophotometer (Fisher Scientific Company LLC, Hanover Park, IL, USA) at 260 nm, procuring a variation of <5%. The standard concentration of the stock solution was calculated by the Beer’s law formula, as shown in Equation (1). This standard concentration was used to generate the standard curve for the HPLC with vitamin D_3_ concentrations of 0.1, 0.5, 1, 5, and 10 µg/mL using 100% methanol HPLC grade (Fisher Scientific Company LLC., Hanover Park, IL, USA) as a dilution solvent and mobile phase.

(1) C (g100 mL)=AE1%

C = concentration in g/100 mL, A = absorbance, $E^{1\%}$ = extinction coefficient 1% in EtOH at 260 nm.

#### 4.5.2. Fortification of Model Fresh Cheese with Vitamin D_3_

To fortify model fresh cheese with vitamin D_3_, crystalline vitamin D_3_ was dissolved in ethanol to reach a final concentration of 2.5 mg/mL [58]. The solution was kept in dark conditions at −27 °C. An aliquot (1.8 mL) of the stock solution was added to the melted anhydrous milk fat to reach a concentration of 2.5 µg of vitamin D_3_/mL of milk [58,59] and mixed using a KA T-25 Digital High-Speed Homogenizer (IKA Works Inc., Wilmington, NC, USA) at 5000 rpm for 2 min. Fortified milk fat was added to the reconstituted skim milk which was used for cheese making. Subdued light conditions were kept during fortification, microfluidization, and cheese making processing. For this study, three experimental units were chosen from Table 3 (P1R1, P1R4, P4R4) since they represented the highest and lowest protein to fat ratios and homogenization pressures. The amount of vitamin D_3_ added was greater than concentrations regularly added to commercial food products due to the small sample size used in the vitamin D_3_ extraction methods explained below.

#### 4.5.3. Traceability of Vitamin D_3_ in Fortified Milk and Cheese

To extract vitamin D_3_ from fortified cheese milk and model fresh cheese, a saponification method was used [58,59,60]. Two grams of sample (milk or cheese) was weighed into a 20 mL glass vial (for cheese samples, a few drops of deionized water were added and mixed in a mortar and pestle); subsequently 8 mL of ethanol (Decon Labs. Inc. King of Prussia, PA, USA); 0.2 g ascorbic acid (Sigma-Aldrich, INC, Milwaukee, WI, USA), a spatletip of pyrogallol ACS 99% (Fisher Scientific Company LLC, Hanover Park, IL, USA); and 3 mL of 50% aqueous potassium hydroxide ACS solution (Fisher Scientific Company LLC, Hanover Park, IL, USA) were added. A nitrogen stream was used to avoid oxidation, and the vial was covered with aluminum foil and left overnight (15 h) under orbital shaking in an incubator (Incu-Shaker mini, Benchmark Scientific, Inc., Edison, NJ, USA) at 95 rpm and maintained at room temperature. After, solvent extraction was performed with 5 mL of hexane and 2 mL of double deionized H_2_O, and the mixture was vortexed and centrifuged (Sorvall ST 16 R, Fisher Scientific Company LLC, Hanover Park, IL, USA) at 2500× *g* for 5 min at 20 °C. Supernatant was collected and evaporated under nitrogen stream, reconstituted in 1 mL of ethanol, and filtered in with a 0.45 µm PTFE (Macherey-Nagel Inc.). Reverse HPLC (Waters e2695 Separation Module, Waters, Milford, MA, USA) with a C18 gravity column (Nucleodur 3 µm, 150 × 4 mm, Macherey-Nagel Inc.) and a photo diode array detector (Waters PDA 996) at 265 nm was used for Vitamin D_3_ quantification. One hundred percent HPLC-grade methanol (Fisher Scientific Company LLC.) was used as mobile phase at a flow rate of 0.5 mL·min^−1^ and an injection volume of 20 µg/mL. Samples were measured in triplicate.

#### 4.5.4. Bioaccessibility of Vitamin D_3_ by In Vitro Digestion

A two phase dynamic in vitro digestion (gastric and intestine) was used to analyze vitamin D_3_ bioaccessibility in the samples of model fresh cheese [61,62,63]. First, the samples were prepared by adding 2 drops of DI water per gram of cheese and were then mixed with a mortar and pestle to aid the extraction. Description of the enzyme cocktail, incubation parameters, and conditions for sample preparation are described in Table 3.

Five grams of sample were placed in a 50 mL conical centrifuge tube. Twenty-seven milliliters of 0.9% saline solution were added followed by 2 mL of gastric phase. The pH was adjusted to 2.0 with 5 M HCL solution. Samples were incubated with orbital shaking at 37 °C and 95 rpm for 1 h. Afterwards, the sample was placed in an ice water bath to decrease the enzymatic action. pH was adjusted to 5.3 with 0.9 M NaHCO_3_ solution. Nine milliliters of intestine phase were added, and pH was adjusted to 7.5 with 2 M NaOH solution. The sample was incubated with orbital shaking at 37 °C and 95 rpm for 2 h.

At the end of the digestion period, samples were centrifuged at 4000× *g* for 20 min at 10 °C. An aliquot was taken from the micellar phase for vitamin D extraction. For vitamin D_3_ extraction, 10 mL of hexane and 4 mL of double deionized H_2_O were added. The mixture was vortexed for 2 min and centrifuged at 2500× *g* for 5 min at 20 °C. Supernatant was collected and extraction was repeated twice. The supernatant was placed under a nitrogen stream and reconstituted with 1 mL 100% MeOH mobile phase. The sample was filtered with a 0.22 µm PTFE (Macherey-Nagel Inc., Bethlehem, PA, USA) and injected in the HPLC. The bioaccessibility was calculated as the amount of recovered vitamin D_3_ after in vitro digestion compared to the amount of vitamin D_3_ found in the cheese matrix.

## Figures and Tables

**Table 1:** Vitamin D_3_ recovery measured throughout processing and after in vitro digestion.

Sample	Cheese Milk after Microfluidization	Cheese Milk after Heat Treatment (82 °C ~ 35 min)	Model Fresh Cheese	Percentage of Vitamin D_3_ Retention in the Model Fresh Cheese	Vitamin D_3_ Recovery after In Vitro Digestion
	µg/mL	µg/mL	µg/g	%	%
P1R1	1.89 ± 0.17 ^a,^*	1.64 ± 0.03 ^a,^*	4.88 ± 0.16 ^a^	52 ± 1.76	64.51 ± 9.96 ^a^
P1R4	1.38 ± 0.11 ^b,^*	1.40 ± 0.11 ^b,^*	4.98 ± 0.28 ^a^	52.8 ± 2.96	41.56 ± 3.63 ^b^
P4R4	0.98 ± 0.19 ^c,^*	0.98 ± 0.17 ^c,^*	4.99 ± 0.31 ^a^	49.3 ± 3.07	27.17 ± 2.90 ^c^

Results are expressed as mean ± standard deviation (*n* = 3). Different letters (a,b,c) within each column denote significant (p<0.05) differences between treatments. * Denotes non-significant differences between the cheese milk after microfluidization and after heat treatment, analyzed by a paired *t*-test.

**Table 2:** Experimental design.

Pressure ^1^ (MPa)	Protein to Fat (P/F) ratio ^2^			
	0.9	1.3	1.7	2
**17**	P1R1	P1R2	P1R3	P1R4
**50**	P2R1	P2R2	P2R3	P2R4
**75**	P3R1	P3R2	P3R3	P3R4
**150**	P4R1	P4R2	P4R3	P4R4

^1^ P = homogenization pressure. ^2^ R = protein to fat (P/F) ratio.

**Table 3:** Enzyme cocktail for in vitro digestion, incubation parameters, and conditions for sample preparation.

Two Phase In-Vitro Digestion	Enzyme Cocktail Solution	Buffer Solution	pH Conditions	Incubation Conditions
**Gastric Phase**	0.9% Saline Solution and 4 g/L pepsin from porcine gastric mucosa in 0.1 M Hydrochloric acid.	5 M HCL	pH = 2.0	Incubation with orbital shaking at 37 °C and 95 rpm for 1 h
**Intestine Phase**	2 g/L pancreatin from porcine pancreas and 12 g/L bile extract in 0.1 M NaHCO_3_	0.9 M solution of NaHCO_3_, 2 M NaOH	Initial pH = 5.3 Final pH = 7.5	Incubation in orbital shaking at 37 °C and 95 rpm for 2 h
